# Plasma interleukin responses as predictors of outcome stratification in patients after major trauma: a prospective observational two centre study

**DOI:** 10.3389/fimmu.2023.1276171

**Published:** 2023-11-23

**Authors:** Matthew Allan Jones, James Hanison, Renata Apreutesei, Basmah Allarakia, Sara Namvar, Deepa Shruthi Ramaswamy, Daniel Horner, Lucy Smyth, Richard Body, Malachy Columb, Mahesan Nirmalan, Niroshini Nirmalan

**Affiliations:** ^1^ Biomedical Research and Innovation Centre, School of Science, Engineering and Environment, University of Salford, Manchester, United Kingdom; ^2^ Critical Care Unit, Manchester University National Health Service (NHS) Foundation Trust (MFT), Manchester, United Kingdom; ^3^ Faculty of Biology, Medicine and Health, School of Medical Sciences, University of Manchester, Manchester Academic Health Science Centre, Manchester, United Kingdom; ^4^ Critical Care Unit, Salford Royal Foundation Trust (SRFT), Salford, United Kingdom; ^5^ Division of Infection, Immunity and Respiratory Medicine, University of Manchester, Manchester, United Kingdom

**Keywords:** trauma, cytokines, interleukin-6, interleukin-10, SOFA, biomarkers

## Abstract

**Background and objectives:**

There is a need to develop objective risk stratification tools to define efficient care pathways for trauma patients. Biomarker-based point of care testing may strengthen existing clinical tools currently available for this purpose. The dysregulation of pro- and anti-inflammatory cytokines in the pathogenesis of organ failure is well recognised. This study was carried out to evaluate whether blood concentrations of IL-6, IL-10, and IL-6:IL-10 ratios in the early stages of the illness are significantly different in patients with worsening organ function.

**Materials and methods:**

In this prospective observational cohort study, plasma concentrations of IL-6 and IL-10 on days 1, 3 and 5 were measured in 91 major trauma patients using a multiplexed cytometric bead array approach. A composite measure of adverse outcome - defined as SOFA ≥ 2 or mortality at 7 days, was the primary outcome. IL-6 and IL-10 concentrations in early samples (days 1, 3 & 5) in patients who developed SOFA ≥ 2 on day 7 were compared against those who did not. Similar composite outcome groups at day 5 and in groups with worsening or improving SOFA scores (ΔSOFA) at days 7 and 5 were undertaken as secondary analyses.

**Results:**

Stratification on day 7, 44 (48%) patients showed adverse outcomes. These adverse outcomes associated with significantly greater IL-6 concentrations on days 1 and 5 (Day 1: 47.65 [23.24-78.68] Vs 73.69 [39.93 – 118.07] pg/mL, *P* = 0.040 and Day 5: 12.85 [5.80-19.51] Vs 28.90 [8.78-74.08] pg/mL; *P* = 0.0019). Similarly, IL-10 levels were significantly greater in the adverse outcome group on days 3 and 5 (Day 3: 2.54 [1.76-3.19] Vs 3.16 [2.68-4.21] pg/mL; *P* = 0.044 and Day 5: 2.03 [1.65-2.55] Vs 2.90 [2.00-5.06] pg/mL; *P <*0.001). IL-6 and IL-10 concentrations were also significantly elevated in the adverse outcome groups at day 3 and day 5 when stratified on day 5 outcomes. Both IL-6 and IL-6:IL-10 were found to be significantly elevated on days 1 and 3 when stratified based on ΔSOFA at day 5. This significance was lost when stratified on day 7 scores.

**Conclusions:**

Early IL-6 and IL-10 concentrations are significantly greater in patients who develop worsening organ functions downstream. These differences may provide an alternate biomarker-based approach to strengthen risk stratification in trauma patients.

## Introduction

Trauma remains a leading cause of morbidity and mortality worldwide, with road traffic accidents accounting for the second highest cause of death in 15-29-year-olds (1). In the United Kingdom (UK) alone, approximately 17,000 major trauma incidents are recorded each year, with accidental falls and road traffic accidents accounting for 85% of these cases (2). Deaths following trauma follows a bimodal distribution - early and delayed. Early mortality following trauma is frequently attributable to the presence of severe head injury, damage to major organs and haemorrhage associated with damage to major vessels, whilst sepsis and multiorgan failure (MOF) are the leading causes of delayed deaths occurring days to weeks post injury (3, 4). Although mortality from major traumatic injury has decreased in recent years, sepsis and MOF remain predominant causes of delayed deaths (5). There is a pressing need to focus on this cohort of patients who deteriorate following admission, with recurrent infections, sepsis, MOF, and death. Data from the Trauma Audit and Research Network (TARN) (6, 7) showed a clear difference in the demography of trauma deaths favouring a shift towards delayed deaths occurring in elderly patients after relatively modest injuries due to the onset of delayed organ failure in trauma victims (6, 7). In this context, understanding and better characterising the pathophysiology of immune-mediated secondary organ failure becomes clinically important.

The inflammatory responses that occur in the context of critical illness, including trauma, play an important role in the development of MOF. These inflammatory processes are initiated by tissue damage, haemorrhage, activation of coagulation cascades and secondary infections that are commonly seen after trauma. The role of pro-inflammatory cytokines in initiating and modulating these inflammatory responses are well recognised (4, 8, 9). In this context, the pathophysiology of trauma closely resembles the pathophysiology of sepsis and is mediated by a dysregulated cascade of immune mediators (9, 10). Plasma concentrations of some of the well characterised pro- and anti-inflammatory cytokines provide a convenient (but potentially crude) window into these interactions (11). Even though a significant body of work has already been done in patients with sepsis to elucidate these pathways, the evidence bases to understand these complex pathways in trauma patients need further characterisation. Understanding these pathways further may help us to stratify patients to different risk groups through point of care testing so that appropriate care pathways may be defined leading to better utilisation of scarce resources.

Preliminary data suggests that the onset and progression to MOF is linked to an imbalance of the systemic pro- and anti-inflammatory responses leading to the overactivity of the latter and hence, an increased susceptibility to sepsis and MOF (4, 12). These dynamic imbalances have been commonly alluded to as Systemic Inflammatory Response Syndrome (SIRS) and/or Compensatory Anti-Inflammatory Response Syndrome (CARS) in medical literature (12). Deciphering the details of this interaction could potentially yield early predictive biomarker profiles for poor clinical outcome in major trauma. Many of these responses mediated by changes in inflammatory molecules, such as cytokines, which are systemically and locally produced following traumatic injury *via* the release of damage associated molecular patterns (DAMPs) and subsequent activation of the innate immune system (13, 14). This release of cytokines has been shown to be significantly elevated after traumatic injury to initiate repair and recovery (12, 15–18).

Of all cytokines previously examined following traumatic injury, IL-6 and IL-10 have shown promise as potential immune biomarkers of patient outcomes. A meta-analysis of 11 publications (775 total patients) by Qiao et al. (19) highlights elevated systemic IL-6 concentrations after 24 hours to potentially predict patient mortality and MOF following traumatic injury, however predictive potential of IL-6 was poor for the development of septic complications. This meta-analysis highlights the predictive potential of IL-6, however there is limited similar evidence for if the same pattern is true for IL-10. Further to this, previous studies investigating the role of IL-6 and to a lesser extent IL-10 in the period following traumatic injury, have shown both IL-6 and IL-10 to correlate strongly with injury severity score (19–23). These studies further support the prognostic potential of both pro- and anti-inflammatory cytokines for patient stratification in major trauma.

These studies however are often limited to examining the concentrations of IL-6 and/or IL-10 at a single time point or within 24 hours of injury (which is ideal for predictive biomarkers), however the temporal profile of IL-6 and IL-10 following major trauma remains uncharacterized. Developing a better understanding of this temporal profile may allow for earlier identification of a compensatory anti-inflammatory response which normally occurs in the days following trauma rather than in the initial 24 hours (24). This study therefore seeks to determine if variations in these profiles are present when stratified based on clinical outcomes characterized by retrospective clinical scoring systems and overall patient outcomes.

Currently, risk stratification in trauma is largely based on retrospective clinical tools such as the injury severity score (ISS), an anatomical scoring system that summarises injury severity based on the most severe injury in six key anatomical areas (25, 26). ISS is based on a wide range of physiological and demographic data, which can only be obtained in hindsight, with injuries used to calculate the score are based on wider diagnostic testing and imaging, hence cannot be used for risk stratification during the acute care phase (27). Furthermore, any scoring system based on physiological derangements can be non-specific as these physiological responses (such as tachycardia, hypotension, tachypnoea etc.) are generic, subject to within and between subject heterogeneity and modified by common medications such as beta blockers. They can be influenced by pain, anxiety, hypovolaemia etc. which can lead to misclassification. There is therefore a strong need to utilise these scoring systems (which are available to clinicians much later in the lifespan of patient care) with early biological mediators (biomarkers) suggestive of dysregulated inflammatory response, to improve risk stratification and prevent misclassification.

This study was undertaken with the following objectives: To characterise the temporal evolution of two prototypical pro- (IL-6) and anti-inflammatory (IL-10) cytokines following major multi-cause traumatic injury. To correlate the temporal and ratiometric changes in cytokine profile (IL-6, IL-10, and IL-6:IL-10) to the clinical phenotypes of injury severity and organ dysfunction determined *via* routinely utilised retrospective clinical scoring systems.

## Materials and methods

### Study design, participants, and setting

A prospective observational cohort study of major trauma patients admitted to Manchester Royal Infirmary, UK and Salford Royal Infirmary, UK between September 2015 and May 2018 were recruited with deferred consent. Participants or a consultee were approached as soon as possible after the injury to confirm consent or assent for inclusion in the study and were free to withdraw at any time. Inclusion criteria were 1) injury severity requiring immediate transfer from the emergency department to the operating theatre or critical care, 2) enrolment within 24 hours of the injury. Principle exclusion criteria were 1) age <18 years, 2) patients on steroids or other immunosuppressive medication 3) sole traumatic brain injury (TBI) as main diagnosis on admission. Routine descriptive data including demographics, injury severity, organ dysfunction and haemorrhage were collected for 7 days following the injury by dedicated research nurses in an anonymised database. Of the 138 patients initially recruited to the study, 47 of these subsequently withdrew consent, resulting in a total of 91 patients available for analysis.

### Sample collection and processing

Blood samples were collected from the participants on the day of injury (D1) and then three (D3) and five (D5) days following the injury. Blood was collected in K_2_EDTA blood vacutainers and transferred to the University of Salford, Manchester, UK, for processing. Plasma was isolated following centrifugation for 10 minutes at 2000 xg. Isolated plasma was stored at -80°C, until required for analysis.

### Interleukin-6 and interleukin-10 cytometric bead array

Cytokine concentrations were quantified using multiplexed cytometric bead array flex sets for IL-6 and IL-10 (Becton, Dickinson, and Company (BD), USA) (28, 29). Plasma samples were analysed in triplicate according to the manufacturer’s instructions. All experiments were conducted using a BD Bioscience FACSVerse flow cytometer (Becton, Dickinson and Company, USA) with data acquired using BD FACSuite (Becton, Dickinson and Company, USA). Mean fluorescence values were converted to concentrations using generated standard curves for each cytokine as described in (30).

### Statistical analysis

The primary outcome was to compare plasma IL-6 and IL-10 levels on admission in two groups based on a composite outcome at 7 days. Composite outcomes were defined as good (SOFA score <2) and poor (SOFA ≥2 or mortality). Secondary outcomes included a comparison of IL-6, IL-10, and concentrations between the two outcome groups at day 5 and a comparison of early cytokine concentrations between two outcome groups defined on the basis of changes in SOFA scores at days 5 and 7 (or ΔSOFA: day seven/five SOFA score - day one SOFA score). For this latter analysis, the outcome groups were defined either as ‘worsening organ failure’ (ΔSOFA ≥0) or ‘Resolving organ failure’ (ΔSOFA <0).

Data are presented as median [interquartiles] and count (%). IL-6 and IL-10 plasma concentrations were analysed using Mann-Whitney *U*-statistics for composite outcomes. Trends over time were analysed using general linear mixed models (GLMM) following log_e_ transformation. Odds ratios (OR) and 95% confidence intervals were estimated using robust logistic regression. Outliers were determined as 1.5 times interquartile range greater than the 3^rd^ quartile or 1.5 times interquartile range less than the 1^st^ quartile. Association or correlation of categorical outcomes was assessed using the Phi Φ statistic for contingency tables. Significance was defined as *P <*0.05 (two-sided) with no corrections for multiple comparisons in this exploratory study. Analyses were performed using GraphPad Prism 9 (GraphPad Inc., La Jolla CA), Stata 16.1, (Stata Corp., College Station TX) and Number Cruncher Statistical Systems 2020 (NCSS) (NCSS Inc., Kaysville UT).

### Ethics approval and consent to practice

All study procedures were approved by the National Research Ethics Committee (REC) South Manchester, UK (REC reference: 15/NW/0262), NHS/HSC Research and Development offices (IRAS ID: 172620) and the ethical committee at the University of Salford (Ethics code: ST1617-17).

## Results

### Summary of patient cohort characteristics

The patient cohort used in this study suffered a broad range of multi-trauma injuries including blunt and penetrating injuries. Specific details of each individual patients’ injuries were not available at the time of analyses. Patient characteristics and baseline measurements at admission are reported in **Table 1**, stratified according to outcome on day 7. Glasgow coma score (GCS), SOFA score and the need for antibiotics or positive-pressure ventilation (IPPV) on day 1 were found to significantly differ between the two groups (**Table 1**). Even though the median ISS were higher in the group showing adverse outcome (27.5 Vs 23.5), this difference did not reach statistical significance. Two patients died in the first 7-days with a mortality rate of 2.2% (95% CI: 0.3–7.7) with a further five patients dying during their hospitalisation resulting in an overall mortality rate of 7.7% (95% CI: 2.2–13.2). Favourable (SOFA <2) and unfavourable (SOFA ≥2) clinical outcomes on or after day 7 occurred in 53 (58.2%) and 38 (41.8%) patients, respectively.

**Table 1:** Patient characteristics and clinical parameters on admission in patients stratified on outcome seven days following trauma.

Variable	All Patients (n = 91)	SOFA <2 on day 7 after admission (n = 53)	SOFA ≥2 on day 7 after admission or Death (n = 38)	Odds Ratio (95% CI)	*P* value
Age (years)	41.5 [29.0-60.8]	39.0 [28.0-53.0]	44.0 [29.5-64.0]	1.01 (0.99-1.03)	0.55
Sex (male)	66 (72.5)	36 (67.9)	30 (78.9)	1.56 (0.58-4.21)	0.38
HR (min^-1^)	99.0 [13.0-112.0]	101.0 [90.0-113.5]	95.0 [76.0-107.0]	0.99 (0.97-1.01)	0.16
SBP (mmHg)	118.5 [98.8-144.0]	110.0 [94.5-147.0]	129.0 [106.0-142.5]	1.01 (0.99-1.02)	0.13
DBP (mmHg)	64.0 [53.0-77.0]	63.0 [53.0-79.5]	66.0 [53.0-77.0]	1.01 (0.98-1.03)	0.50
GCS	15.0 [6.0-15.0]	15.0 [14.0-15.0]	10.0 [3.0-15.0]	0.84 (0.75-0.95)	0.003**
FiO_2_	0.30 [0.21-0.52]	0.31 [0.21-51.5]	0.25 [0.21-0.65]	1.00 (0.98-1.02)	0.94
PF ratio	31.4 [7.3-49.4]	39.2 [21.6-58.0]	10.3 [3.8-34.2]	0.99 (0.98-1.01)	0.99
WCC (x10^9^ L^-1^)	14.4 [10.0-19.0]	13.7 [10.0-18.8]	15.4 [10.5-19.8]	1.02 (0.99-1.04)	0.19
CRP (mg L^-1^)	18.0 [6.0-60.5]	19.0 [5.0-58.0]	17.0 [6.8-72.0]	1.00 (0.99-1.01)	0.29
Lactate (mmol L^-1^)	2.0 [1.4-3.7]	1.8 [1.2-3.4]	2.9 [1.7-4.8]	1.20 (0.90-1.59)	0.22
ISS	25.0 (9.0-41.0]	23.5 [16.0-29.0]	27.5 [15.5-38.75]	1.04 (1.0-1.08)	0.062
APACHE II	9.0 [6.5-12.0]	9.0 [7.0-11.0]	9.5 [3.3-14.0]	1.05 (0.88-1.25)	0.60
SOFA	4.5 [1.0-9.0]	3.0 [1.0-5.0]	8.0 [4.0-10.0]	1.25 (1.10-1.42)	0.001**
Antibiotics on admission	62 (68.1)	41 (77.4)	21 (55.3)	0.36 (0.15-0.90)	0.022*
IPPV on admission	42 (46.2)	19 (35.8)	23 (60.5%)	2.74 (1.16-6.51)	0.022*

Data has been stratified according to SOFA scores seven days post trauma and presented as median [interquartiles] and count (%). Odds ratio is presented with 95% confidence interval (CI). Statistical significance was determined using Mann-Whitney U tests *P < 0.05,**P <0.01. APACHE, Acute Physiology and Chronic Health Evaluation; CRP, C-Reactive protein; DBP, Diastolic blood pressure; GCS, Glasgow coma score; FiO2, Fraction of inspired oxygen, ISS, Injury severity score; IPPV, Intermittent positive pressure ventilation; SBP, Systolic blood pressure; SOFA, Sequential Organ Failure Assessment; WCC, White blood cell count.

### Cytokines and outcomes

**Figure 1:**
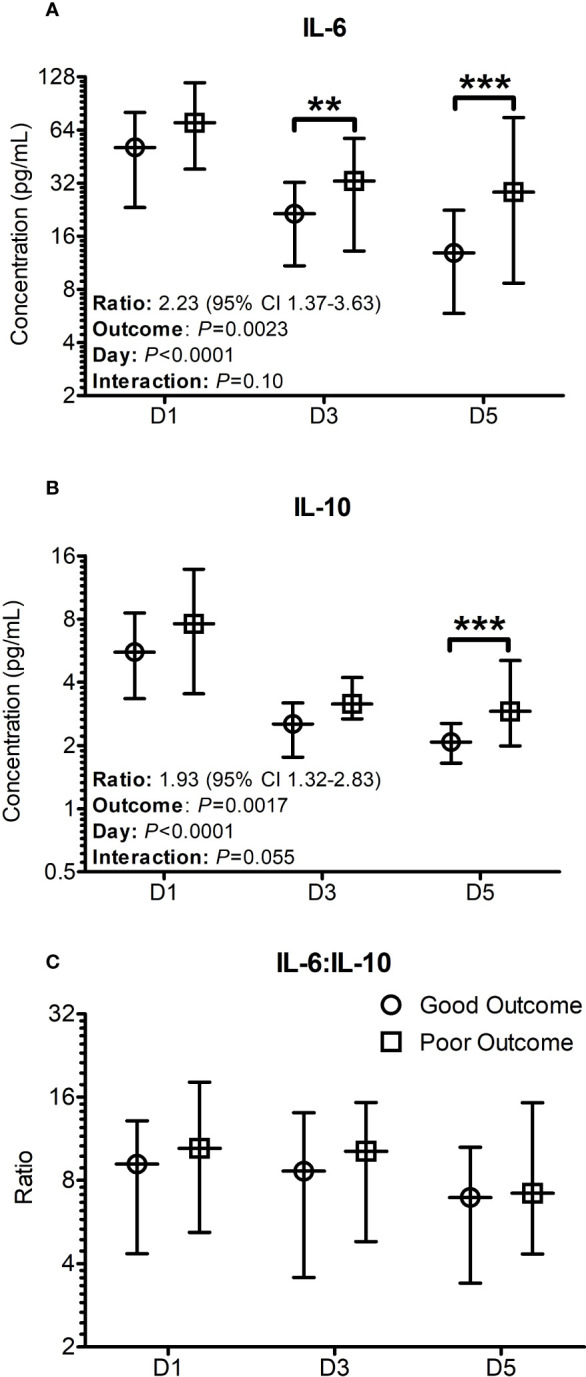
Cytokine profiles significantly differ when stratified based on outcomes seven days post trauma. Patients were stratified based on good and poor outcomes based on their SOFA scores seven days post trauma. Good outcome was classified as a SOFA score of < 2 (Circles on all panels), whilst poor outcome was classified as a SOFA score ≥ 2 (Squares on all panels) seven days post traumatic injury or death. **(A)** The IL-6 levels on day 3 and day 5 were significantly greater in poor outcome patients than good outcome patients at day 7. **(B)** The IL-10 levels on day 5 was significantly greater in patients with a poor outcome. **(C)** The profile of the IL-6:IL-10 ratio was found to not significantly differ between the groups. All data presented as median [Interquartiles]. Statistical significance was determined using general linear mixed models for repeated measures and Bonferroni corrections. n = 91 for D1 and D5, n = 52 for D3. **P <0.01, ***P <0.001.

When the analysis was performed using the general linear mixed models for repeated measures with Bonferroni corrections, the differences in IL-6 concentrations between the two groups were significantly lower in the good outcome patients on days 3 (*P* = 0.009) and 5 (*P* < 0.001) (**Figure 1A**), whereas the differences in IL-10 concentrations were only significantly lower on day 5 (*P* < 0.001) only (**Figure 1B**). The differences between the two groups in respect to the median cytokine concentrations were more pronounced on Day 5 with significance levels reaching *P* < 0.001, even though there was still considerable overlap in the confidence intervals (**Figure 1**). Over the 5 days, the odds ratio of IL-6 between the two groups was found to be 2.23 (95% CI: 1.37-3.63, *P* = 0.0023) compared to 1.93 (95% CI: 1.32-2.83, *P* = 0.0017) in the case of IL-10 (**Figures 1A, B**). Both IL-6 and IL-10 were found to significantly differ in a time (IL-6: *P* = 0.0023; IL-10: *P* = 0.0017) and outcome (Both IL-6 and IL-10: *P <*0.0001) dependent manner. Plots for the decay of cytokine concentrations after the initial rise showed that the decay in IL-6 concentrations were more consistent with a unimodal peak at day 1. The decay in IL-10 concentrations was more complex suggestive of at least a bimodal distribution. No significant differences were seen in relation to IL-6:IL-10 ratios at any time point examined (**Figure 1C**), with significant variation observed within the patient population examined.

When patients were stratified into groups based on greater severity of organ dysfunction (SOFA ≥ 5 or SOFA < 5) at Day 7 (**Supplementary Figure 1**), Day 1 IL-6 concentration was found to be significantly elevated (50.9 [25.9-79.4] (n = 58) Vs 82.8 [41.5-123.0] pg/mL, *P* = 0.0217) in patients with worsened organ function based on their SOFA score at Day 7. No significant differences were observed when the same stratification was performed based on Day 5 SOFA scores.

Correlation analysis of ISS with measured patient outcomes revealed no significant relationship between ISS and day one SOFA socres (R = -0.064, *P* = 0.671), IL-6 levels (R = -0.116, *P* = 0.436), IL-10 levels (R = 0.064, *P* = 0.673), and the IL-6:IL-10 ratio (R = -0.246, *P* = 0.099) (**Supplementary Figure 2**).

**Figure 2:**
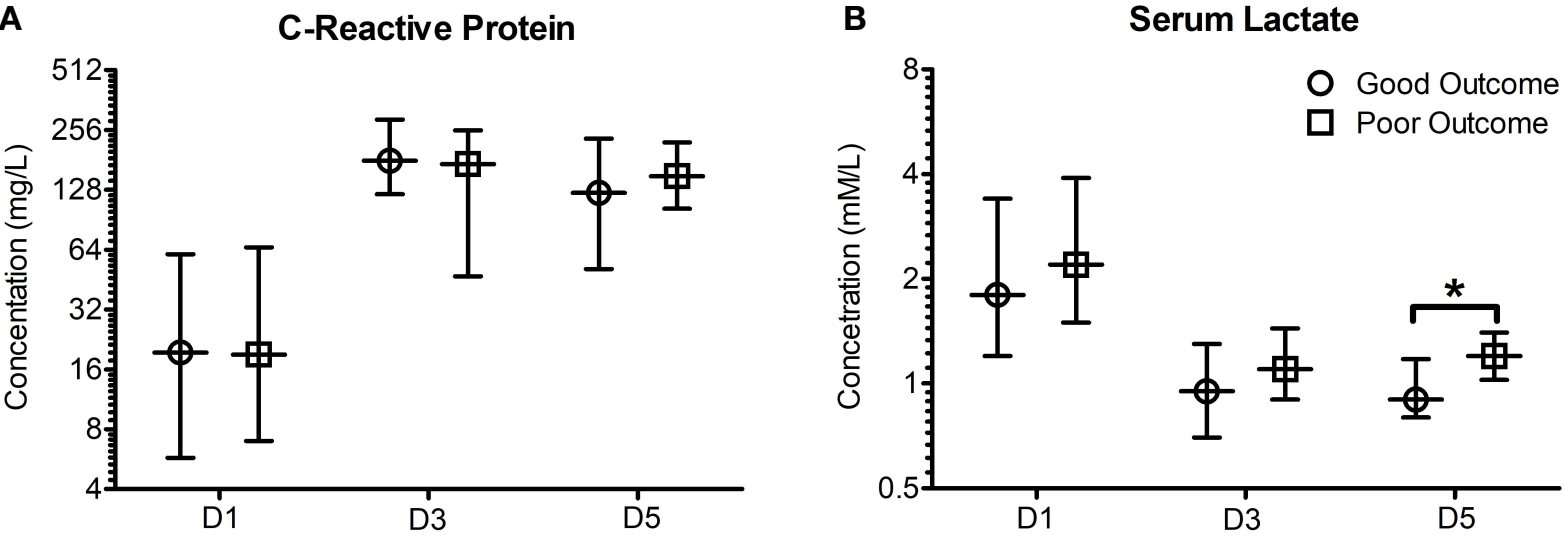
Early (Day 1, 3 and 5) C-reactive protein and serum lactate poorly predict patient outcome when stratified based on outcome seven days post trauma. Patients were stratified based on good and poor outcomes based on their SOFA scores seven days post trauma. Good outcome was classified as a SOFA score of <2, whilst poor outcome was classified as a SOFA score ≥2 five days post traumatic injury or death. **(A)** C-reactive protein concentrations were found to not significantly differ in good and poor outcome patients five days following traumatic injury. **(B)** Serum lactate concentrations were found to be significantly elevated in poor outcome patients five days following traumatic injury (*P* = 0.045). All data presented as median [Interquartiles]. Statistical significance was determined using Mann-Whitney U tests. n = 35-43 for CRP, n = 28-74 for serum lactate. *P <0.05.

Neither the clinical scores such as ISS and APACHE scores, nor the more frequently used biomarkers such as C-Reactive protein (CRP) at days 1, 3 or 5 were significantly different between the two primary outcome groups. The relative distributions of these biomarkers between the two primary outcome groups are shown in **Figure 2**. Only serum [Lactate] at day 5 was found to be significantly different between the outcome groups (0.9 [0.8-1.175] Vs 1.2 [1.025-1.4] mM/L, n = 28, *P* = 0.045).

Worsening organ function as reflected by change in SOFA scores over time (ΔSOFA, the relative difference between days 7 and 1) was observed in 31 of 85 patients (36.47%). No statistically significant differences in IL-6, IL-10, IL-6:IL-10, CRP, and serum [Lactate] was observed in relation to worsening organ function (**Supplementary Figures 3, 4**). Similar analyses as above using clinical outcomes on day 5 are shown in **Supplementary Table 1** and **Supplementary Figures 5**–**8**. These analyses identified similar patterns to that observed when patients were stratified based on outcome at day 7.

**Figure 3:**
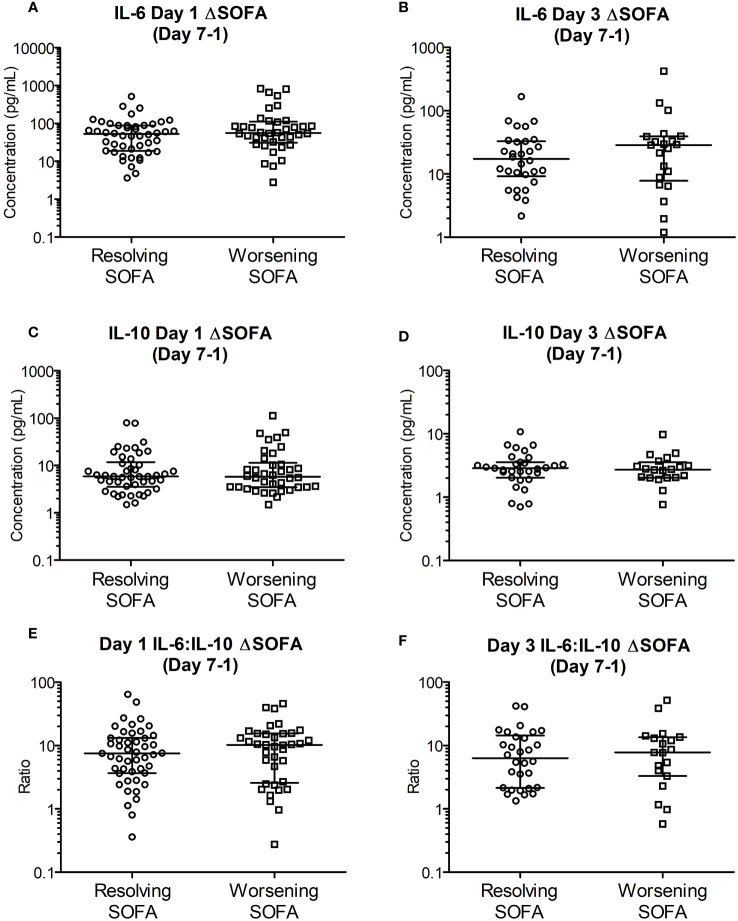
Early (day 1 and 3) cytokine levels are unable to differentiate between deteriorating and resolving SOFA scores in patients stratified seven days post trauma. Patients were retrospectively categorized according to their ΔSOFA, a calculated change in SOFA value (SOFADay7 – SOFADay1 score), creating two patient groups: ΔSOFA <0 (Resolving condition) and ΔSOFA ≥0 (Worsening condition). **(A, B)**. IL-6 levels on day one (52.96 [16.58-88.36] Vs 56.03 [34.64-103.56] pg/mL; n = 85, *P* =0.295) and day three (17.37 [9.18-32.94] Vs 28.58 [8.83-38.94] pg/mL; n = 52, *P* =0.485) did not differ between the two groups. **(C, D)**. IL-10 levels on days one (5.87 [3.71-10.82] Vs 5.77 [3.51-10.47] pg/mL; n = 85, *P* =0.886) and day three (2.86 [2.22-3.51] Vs 2.72 [2.03-3.29] pg/mL; n = 52, *P* =0.714) were also not significantly different between the two groups. **(E, F)**. IL-6:IL-10 on days one (7.51 [3.78-13.14] Vs 10.26 [2.68-15.38]; n = 84, *P* =0.552) and day three (6.35 [2.16-13.54] Vs 7.80 [3.68-13.27]; n = 49, *P* =0.862) were not significantly different between the two groups. All data expressed as median [Interquatiles]. Statistical significance was determined using Mann-Whitney U tests.

**Figure 4:**
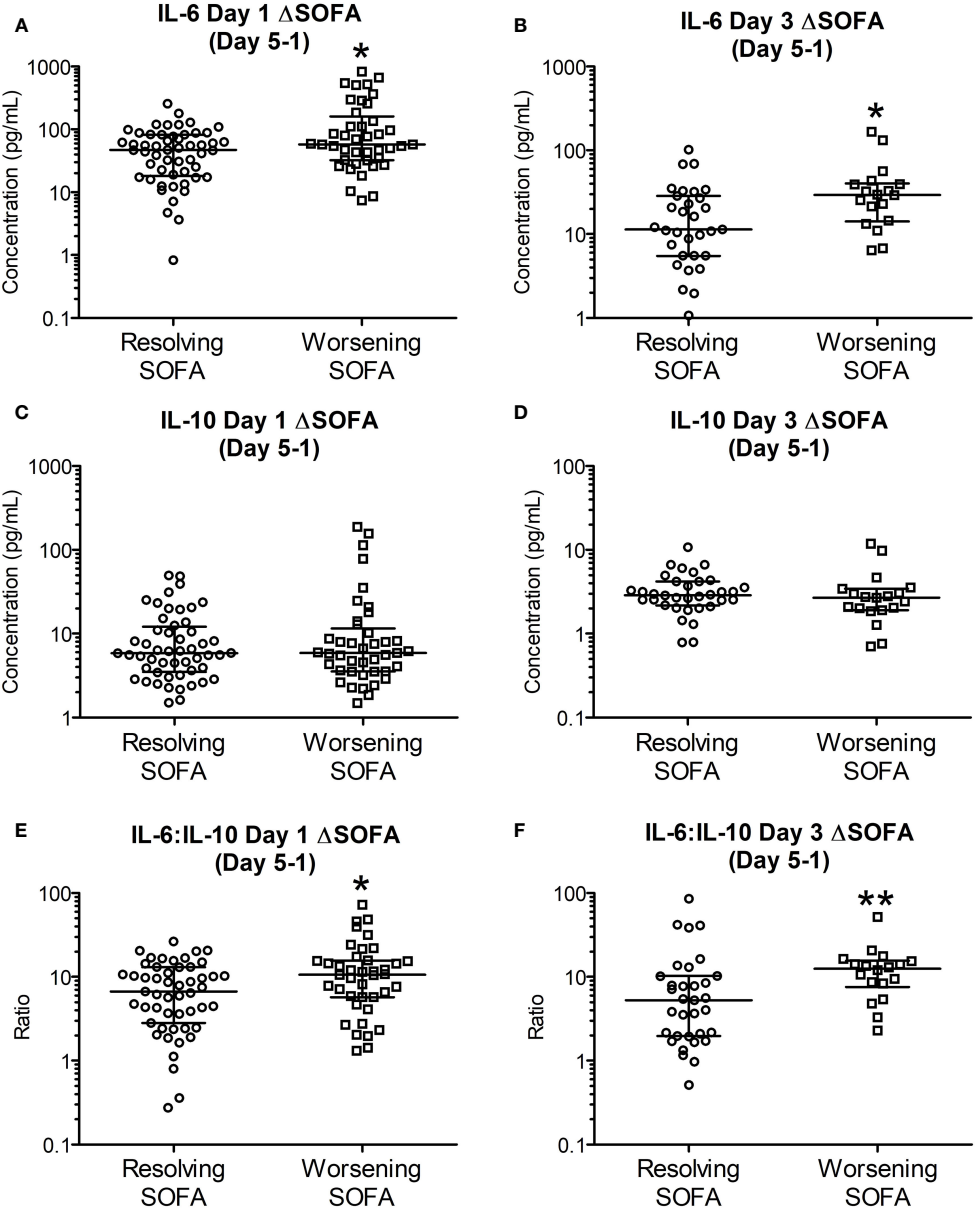
Early (day 1 and 3) IL-6 and IL-6:IL-10 levels are significantly elevated in patients with worsening SOFA scores five days post trauma. Patients were retrospectively categorized according to their ΔSOFA, a calculated change in SOFA value (SOFADay5 – SOFADay1 score), creating two patient groups: ΔSOFA <0 (Resolving condition) and ΔSOFA ≥0 (Worsening condition). **(A, B)**. IL-6 levels on day one (47.01 [19.00-82.01] Vs 57.78 [32.84-136.08] pg/mL, n = 89, *P* =0.030) and day three (11.37 [5.51-27.70] Vs 29.38 [16.21-39.34], n = 51, *P* =0.010) were significantly elevated in patients with worsening SOFA scores. **(C, D)**. IL-10 levels on days one (5.85 [3.59-11.38] Vs 5.87 [3.54-10.19] pg/mL; n = 89, *P* =0.958) and day three (2.88 [2.26-4.08] Vs 2.69 [1.96-3.26] pg/mL; n = 51, *P* =0.278) were not significantly different between resolving and worsening patients. **(E, F)**. IL-6:IL-10 ratios on days one (6.71 [3.22-12.10] Vs 10.26 [5.77-15.54]; n = 89, *P* =0.032) and day three (5.25 [1.98-10.21] Vs 12.48 [8.42-15.02]; n = 51, *P* =0.006) were significantly elevated in patients with worsening SOFA scores. All data expressed as median [Interquatiles]. Statistical significance was determined using Mann-Whitney U tests. *P <0.05, **P <0.01.

IL-6, IL-10 and IL-6:IL-10 for the two groups based on ΔSOFAs at days 5 and 7 after removing the outliers (n = 2-4, justified as described in section 3.4) are shown in **Figures 3**, **4**. The common clinical biomarkers of CRP and serum Lactate were unable to differentiate between resolving and worsening day 5 SOFA scores (**Supplementary Figure 9**).Whilst day 1 and day 3 IL-6 and IL-6:IL-10 were significantly greater in patients who showed worsening of SOFA scores between day 5 and day 1, there were no significant differences in IL-10 (**Figure 4**). Similar differences were not observed between the two ΔSOFA groups when stratified at day 7 (**Figure 3**).

## Discussion

In this preliminary study we have shown that there is a clear demonstrable temporal cytokine response during the early stages following major trauma. We have also shown in trauma patients, that the early plasma concentrations of two prototypical cytokines involved in inflammatory processes (IL-6 and IL-10) are significantly greater in patients who then proceed develop clinical phenotypes of worsening organ dysfunction and SOFA scores. This highlights that the balance of immune mediators may play an important role for the development of predictive biological markers for patient stratification within the ICU. Early identification of high-risk patients provides a potential window during which interventions aimed at preventing the evolution of organ failure (invasive monitoring, close nursing care etc) may be commenced to those most at risk and thereby optimise the utilisation of scarce resources. It is recognised that these cytokines render themselves well for point of care testing and thereby are ideal targets for bedside diagnostic tests that may be built into risk stratification algorithms. Despite there being statistically significant differences between groups that develop organ dysfunction and those who do not, our study shows a wide scatter in the distribution of the concentrations of both cytokines implying that multiple factors may be involved in the regulation of these responses. It is therefore likely that even though a biomarker-based approach to risk stratification may be used to improve the accuracy of clinical decision-making, they are unlikely to replace a traditional, clinician lead, multi-dimensional assessment of a trauma victims. Further larger clinical trials are needed to determine the sensitivity and specificity of a new combined approach utilising clinical criteria supported by point of care testing of cytokines, as a part of risk stratification procedures.

In the study we have used mortality and a SOFA score of ≥2 on day 7 as indicative of an adverse clinical outcome. In established ICU patients it is known that SOFA scores have a linear relationship with ICU mortality (31). As the score represents a composite score derived across six domains - each of which may be scored between 0 (normal) to 4 (severe organ failure), the maximum possible score is 24 (32). Therefore, the threshold of ≥2 represents the lower end of the spectrum involving mild dysfunction affecting one or more organ systems. This threshold on day 7 was selected by the study team as the main outcome measure, as the study was aimed at identifying patients who were clinically deteriorating, but still amenable to clinical interventions. Many of the patients categorised into the adverse outcome group therefore survived their injury and were successfully discharged from hospital. The fact that they were in an intensive care unit is likely to have contributed to the timely recognition and reversal resulting in favourable outcomes. The finding that even at such a low threshold, there were significant differences in cytokine profiles is encouraging when considering the potential value of these two cytokines as suitable targets for developing rapid point of care tests.

This low threshold also could potentially explain why our data shows a relatively wide scatter (represented by the wide inter-quartile intervals) and overlap between groups despite reaching statistical significance. It is therefore possible that by setting the threshold higher to capture patients who are more unwell it may be possible to reduce this overlap, thus better demonstrating the potential value of these cytokines in risk stratification. In the patient cohort examined as a part of this study, there were a limited number of patients who remained at higher clinical outcome thresholds than those selected. Therefore, analysis regarding the potential of IL-6 and IL-10 to stratify these more clinically severe patients would have been statistically underpowered and patterns may not be recognizable. The future expansion and long-term continuation of this study would allow for increased statistical powering of these more severe trauma cases and allow for greater stratification options dependent on the severity of traumatic injury.

Our study found that there was no significant relationship between the retrospective scoring system, injury severity score, and the patient cohorts SOFA scores, and cytokine concentrations (**Supplementary Figure 2**). Whilst ISS is a common scoring system used to quantify trauma severity, it is highly retrospective and utilses an Abbreviated Injury Scale coding system based on identified injuries, many of which may not be realized until weeks or months after the initial trauma (33). In many locations globally it is not practically possible to utilise ISS as a clinical tool during patient stays within the ICU and is more commonly used as a retrospective epidemiological tool calculated after patient discharge when all injuries are known (34). This is the case for the North West United Kingdom hospitals where the study was conducted. It has also been reported that whilst ISS is the “gold standard” scoring system for trauma, it may lack sensitivity and specificity to differentiate between patient cohorts, specifically in patient cohorts from developed countries and in cohort sizes less than 100 patients (34). As both of these parameters are present in our study, this could explain why our patient cohort shows no significant relationships of differences when stratified based on ISS, and highlights the need for further expansion of the study.

The role of cytokines in the evolution of organ dysfunction has been studied extensively in patients with sepsis. Comparatively similar longitudinal studies in trauma patients are few and far between. Whilst many different cytokines have been previously examined following traumatic injury (22, 35, 36), there is limited further research to corroborate these findings. Of all cytokines previously examined, IL-6 and IL-10 have been highlighted to be of great potential in determining injury severity following trauma. Multiple studies have shown both IL-6 (20, 22, 37) and IL-10 (37, 38) concentrations to be aberrantly elevated in the period following trauma. However, limited examination into their diagnostic or prognostic potential for long term traumatic injury outcome and organ dysfunction has been conducted (37, 39, 40). Our study is unique as we have undertaken repeated measurements of these two key pro- and anti-inflammatory cytokine profiles and hence have determined the temporal evolution of these profiles between two distinct clinically relevant cohorts as determined by post discharge clinical scoring systems. These profiles offer the advantage of evaluating the temporal evolution of cytokine levels, allowing for a greater understanding of disease pathophysiology and the identification of how aberrant cytokine expression/production over time is associated with patient prognosis and outcomes.

An extensive body of research reported previously has also highlighted the potential of IL-6 in particular as a biomarker of traumatic injury severity (20, 37, 39, 41–43) and clinically important outcomes such as; multi-organ dysfunction (37, 38, 40, 44, 45), sepsis (46) and mortality (20, 37). A study by Frink et al. (45) showed that IL-6 was found to be significantly higher in patients with MODS compared to those without organ dysfunction in 143 trauma patients. Additionally, they identified that early plasma IL-6 concentrations could identify patients who were at higher risk of developing MODS with high specificity but low sensitivity (45). The concentrations of IL-6 in our study were significantly greater in groups with worsening organ functions on day 7 (**Figure 1**) and day 5 (**Supplementary Figure 3**) and in patients who were showing worsening organ functions between days one and five (**Figures 4A, B**). IL-6:IL-10 ratio is considered to be an index showing the balance between pro and anti-inflammatory pathways (37, 42).

In our analyses the difference in this ratio reached statistical significance only in the ΔSOFA outcome groups on day 5 (**Figures 4E, F**) after excluding 2-4 outliers. These observed differences were not evident against the ΔSOFA outcome groups on day 7 (**Figure 3**). These discrepancies are attributable to the multitude of factors involved in the regulation of particularly IL-10 – a key counter regulatory cytokine which is produced by the body to quench the pro-inflammatory pathways activated by trauma (47). Unlike IL-6 levels which were significantly different on day 3 itself (**Figure 1**), difference in IL-10 concentrations between the two outcome groups reached statistical significance only on day 5. This observation supports the role of IL-10 as a counter regulatory cytokine, with the relevant plasma concentrations lagging that of IL-6 which mediates the pro-inflammatory responses that trigger IL-10 production. Compared to IL-6 levels, the decline in IL-10 concentrations did not follow a single unimodal decline (**Figure 1**) suggesting further that the regulation of IL-10 is more complex and hence plasma concentrations are potentially less predictable. IL-10 (and other counter regulatory cytokines) potentially contribute to immunosuppression thereby making these patients more vulnerable to hospital acquired infections at this stage of their illness.

The finding of significantly greater concentration of plasma [lactate] on day 5 (**Figure 2**), a common association with sepsis, is a very pertinent and interesting finding in this context. The production and plasma concentrations of counter regulatory cytokines such as IL-10 are therefore subject to multiple factors including fluid resuscitation, antibiotics, secondary infections, and other supportive care. The cumulative impact of these therapeutic interventions are likely to be manifest by day 7 and hence, in the present study we have explored the relationships between plasma [cytokine] on clinical outcomes at day 5 and day 7. **Figure 4** shows that IL-6 and IL-6:IL-10 ratios were significantly greater in the group showing deterioration in the SOFA score between days 1 and 5. These differences are not evident at ΔSOFA Day 7 as the effects of interventions become more manifest making IL-10 concentrations more variable. Our study further develops the existing body of data in this area by showing that the early absolute values as well as the temporal changes in IL-6 and IL-10 during the first few days (days 1 and 3) after injury may be an early marker of organ dysfunction (**Figures 3**, **4**). More importantly these differences are evident early in the course of the illness (days 1-3) where potential interventions aimed at reversing these processes are possible.

Our study did not show any differences in CRP or serum [lactate] between the two outcome groups on days 1-3. While no significant differences were seen in CRP on day 5, plasma [lactate] was higher in the group developing worsening organ functions (**Figure 2**). Serum [lactate] in particular has been highlighted as a useful predictor of clinical outcome in trauma patients (48–51). These studies, however, were almost uniformly undertaken in the emergency departments and hence would have been a marker of severity of shock and the quality of care in the pre-hospital environment. Our patients were recruited after their initial management in an A&E department where they would have received adequate resuscitation and reversal of shock/tissue hypo perfusion. It is therefore not surprising that there was a poor association between serum [lactate] on days 1 and 3 and organ dysfunction on day 7 (**Figure 2**). Serum [lactate] levels on day 5 on the other hand were significantly greater (**Figure 2**), perhaps with the onset of sepsis or other factors that may affect tissue perfusion/metabolism, in the group showing greater organ dysfunction. CRP, usually a marker of inflammation or sepsis, was not significantly different between the two groups at any point in our study.

An important limitation of the present study needs emphasis. As this was an observational study, the effect of treatment remains an important confounding factor. Secondary infections, use of antibiotics, fluid management and ventilation associated lung injury (VILI) are all examples of potential modifiers of early cytokine responses and may account for the relatively wide confidence intervals in our data and considerable overlap between groups. Despite these confounders, we have shown that significant differences do exist between cohorts of patients who undergo worsening of organ functions or not. Larger longitudinal studies are required to determine the value of these early cytokine responses in predicting clinical outcomes and if their incorporation into decision-making algorithms is a viable option for improved risk stratification.

## Conclusion

Our study highlights the potential of IL-6, IL-10, and IL-6:IL-10, as potential biomarkers for predicting negative patient outcomes and deterioration in patients after major trauma. The study shows that even though IL-6 and IL-10 concentrations were greater in the adverse clinical outcome groups, the temporal evolution of IL-6 was more consistent for potential clinical applications.

## Data availability statement

The raw data supporting the conclusions of this article will be made available by the authors, without undue reservation.

## Ethics statement

The studies involving humans were approved by National Research Ethics Committee (REC) South Manchester, UK (REC reference: 15/NW/0262), the NHS/HSC Research and Development offices (IRAS ID: 172620), and the Ethical committee at the University of Salford (Ethics code: ST1617-17). The studies were conducted in accordance with the local legislation and institutional requirements. The participants provided their written informed consent to participate in this study.

## Author contributions

MJ: Data curation, Formal Analysis, Investigation, Methodology, Writing – original draft, Writing – review & editing. JH: Conceptualization, Investigation, Methodology, Project administration, Resources, Supervision, Writing – original draft. RA: Writing – original draft, Data curation, Investigation, Methodology. BA: Writing – original draft, Data curation, Funding acquisition, Investigation, Methodology. SN: Formal Analysis, Writing – original draft, Writing – review & editing. DR: Writing – original draft, Investigation, Methodology. DH: Methodology, Project administration, Resources, Supervision, Writing – original draft, Investigation. LS: Conceptualization, Supervision, Writing – original draft. RB: Conceptualization, Funding acquisition, Investigation, Project administration, Resources, Supervision, Writing – original draft. MC: Conceptualization, Formal Analysis, Writing – original draft. MN: Conceptualization, Project administration, Resources, Supervision, Writing – original draft, Writing – review & editing. NN: Conceptualization, Funding acquisition, Investigation, Methodology, Project administration, Resources, Supervision, Writing – original draft, Writing – review & editing.
