# Supplementary material for: Plasma interleukin responses as predictors of outcome stratification in patients after major trauma: a prospective observational two centre study
Source: Front Immunol. 2023 Nov 23;14:1276171. doi: 10.3389/fimmu.2023.1276171 (PMC10702136; doi:10.3389/fimmu.2023.1276171)
Supplement: Supplementary file 1 [file DataSheet_1.docx]

Supplementary Material

# Supplementary Figures and Tables

## Supplementary Figures


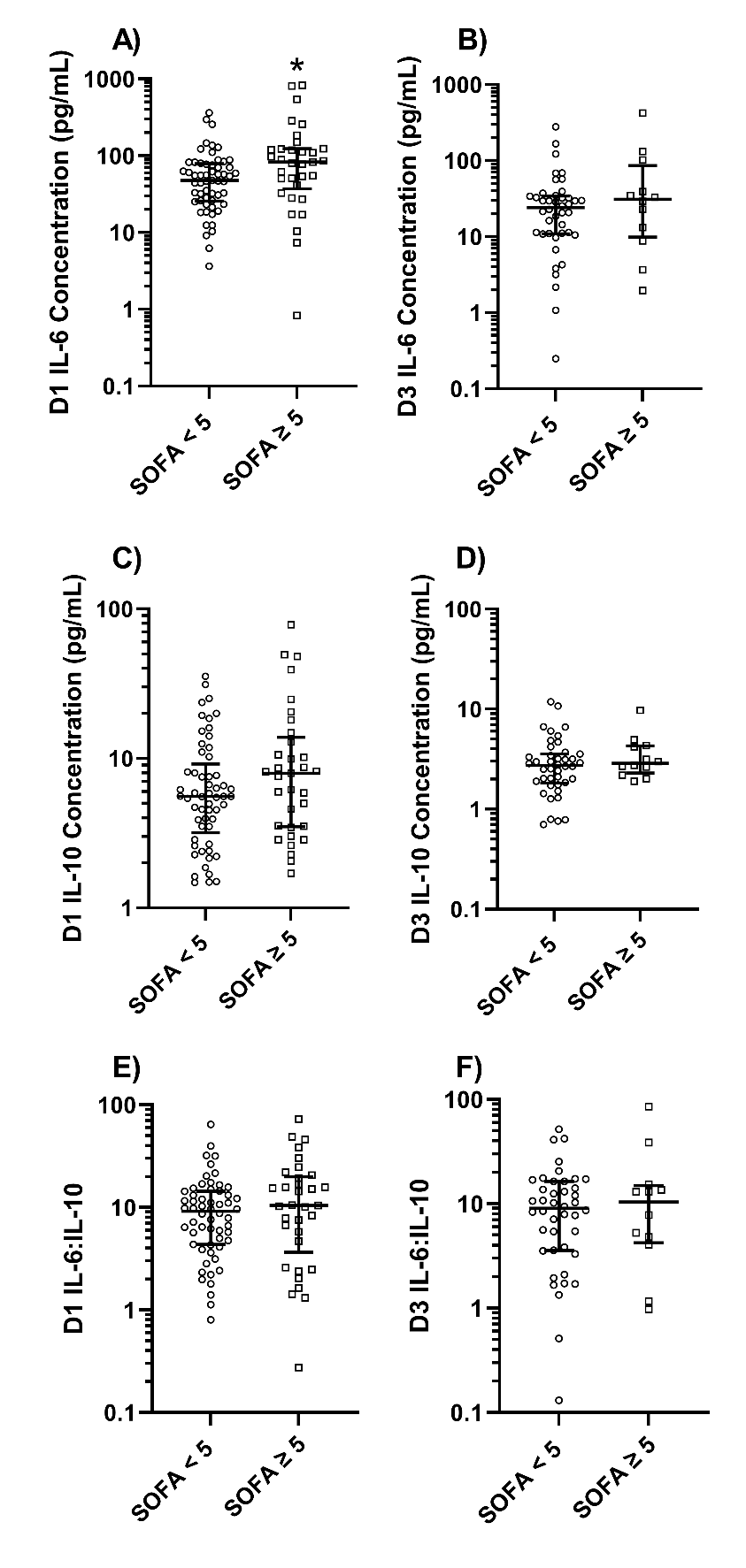
**Supplementary Figure 1.** **Cytokine profiles significantly differ when stratified based on outcomes five days post trauma**.

Patients were stratified based SOFA scores seven days post trauma. Patients were stratified based on their SOFA scores seven days post traumatic injury with a SOFA score of <5, and a SOFA score ≥5 or death being the two outcome groups. **A.** The IL-6 levels on day 1 were found to be significantly elevated in patients with SOFA scores ≥5 than those with SOFA score of <5. **B.** The IL-6 levels on day 3 did not significantly differ between patient outcome groups. **C & D.** The IL-10 levels on day 1 and day 3 did not significantly differ between patient groups **E & F.** The profile of the IL-6:IL-10 ratio was found to not significantly different between the groups. All data presented as median [Interquartiles]. Statistical
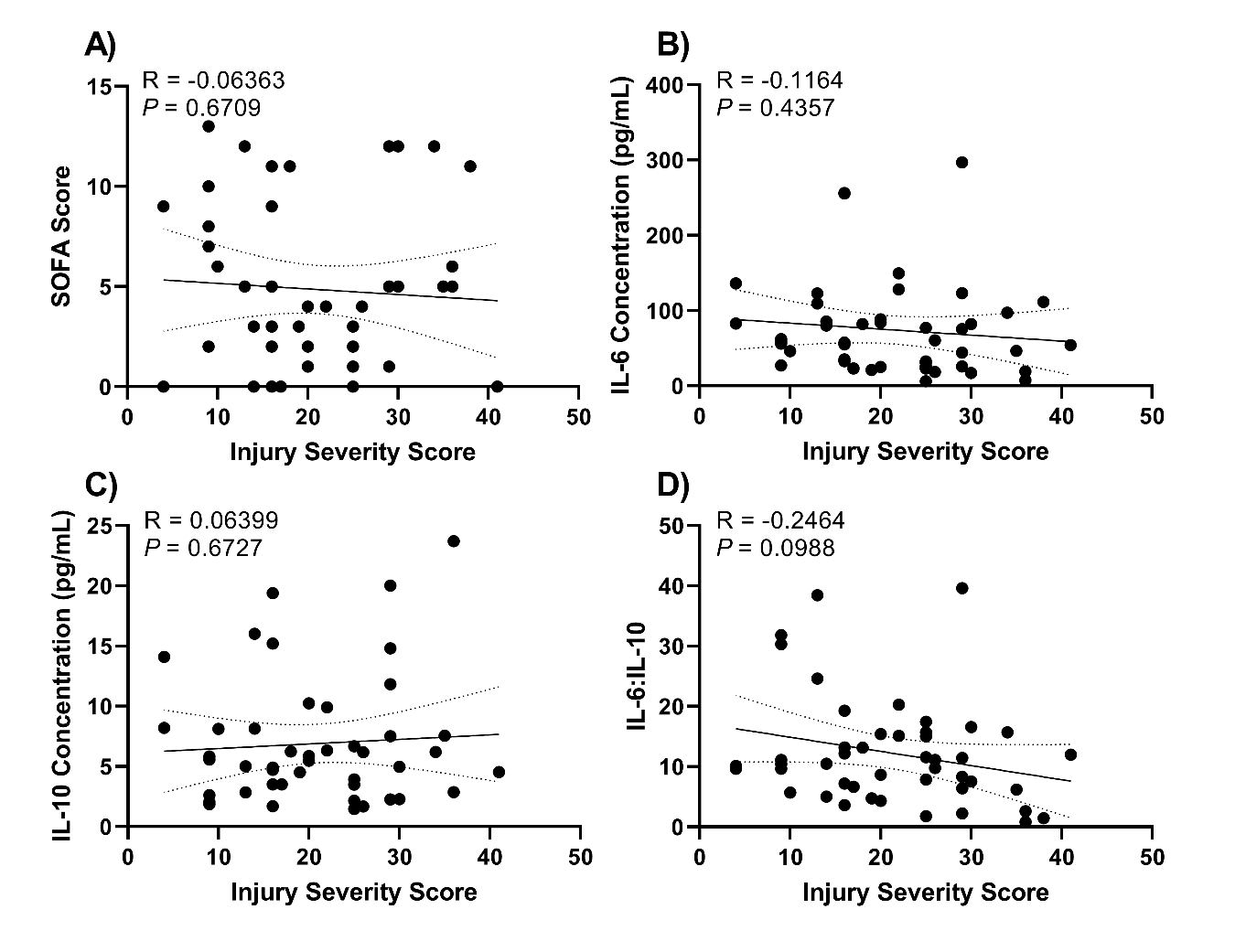
significance was determined using Mann-Whitney U tests. * = P <0.05.

**Supplementary Figure 2.** **Patient cohort day one SOFA scores and cytokine concentrations do not significantly correlate to ISS.**

**A.** The correlation of ISS to day one SOFA scores reveals no significant relationship in the patient cohort. **B.** The correlation of ISS to day one IL-6 levels reveals no significant relationship in the patient cohort. **C.** The correlation of ISS to day one IL-10 levels reveals no significant relationship in the patient cohort. **D.** The correlation of ISS to the day one IL-6:IL-10 ratio reveals no significant relationship in the patient cohort. n = 47 for all panels Dashed lines indicate 95% confidence intervals for the fitted linear regression.

**
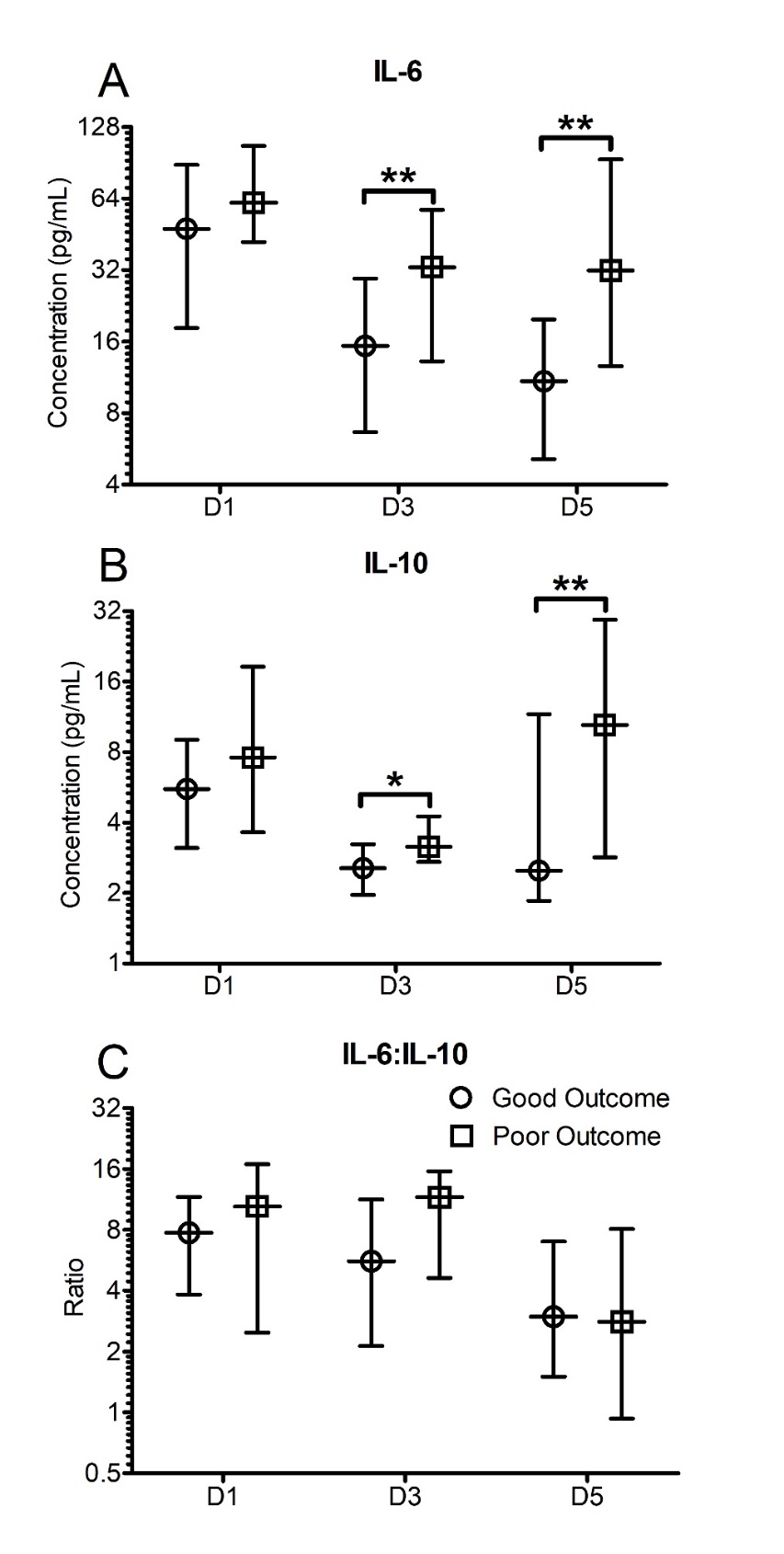
Supplementary Figure 3.** **Cytokine profiles significantly differ when stratified based on outcomes five days post trauma**.

Patients were stratified based on good and poor outcomes based on their SOFA scores five days post trauma. Good outcome was classified as a SOFA score of <2, whilst poor outcome was classified as a SOFA score ≥2 five days post traumatic injury or death. **A.** The IL-6 levels on day 3 and day 5 were significantly greater in poor outcome patients than good outcome patients at day 5. **B.** The IL-10 levels on day 3 and day 5 were significantly greater in patients with a poor outcome on day 5 than in patients who had a good outcome. **C.** The profile of the IL-6:IL-10 ratio was found to not significantly different between the groups. All data presented as median [Interquartiles]. Statistical significance was determined using Mann-Whitney U tests. * = *P* <0.05, ** = *P* <0.01.


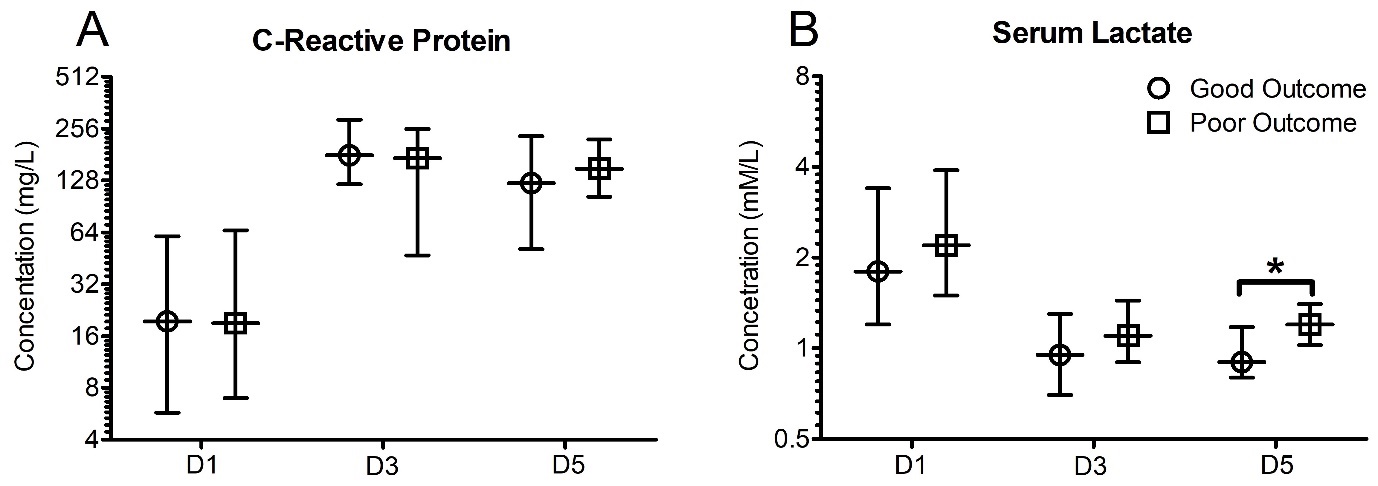


**Supplementary Figure 4. Early (Day 1, 3 and 5) C-reactive protein and serum lactate poorly predict patient outcome when stratified based on outcome seven days post trauma.**

Patients were stratified based on good and poor outcomes based on their SOFA scores seven days post trauma. Good outcome was classified as a SOFA score of <2, whilst poor outcome was classified as a SOFA score ≥2 five days post traumatic injury or death. **A.** C-reactive protein concentrations were found to not significantly differ in good and poor outcome patients five days following traumatic injury. **B.** Serum lactate concentrations were found to be significantly elevated in poor outcome patients five days following traumatic injury (*P* = 0.045). All data presented as median [Interquartiles]. Statistical significance was determined using Mann-Whitney U tests. n = 35-43 for CRP, n = 28-74 for serum lactate. * = *P* <0.05.

**
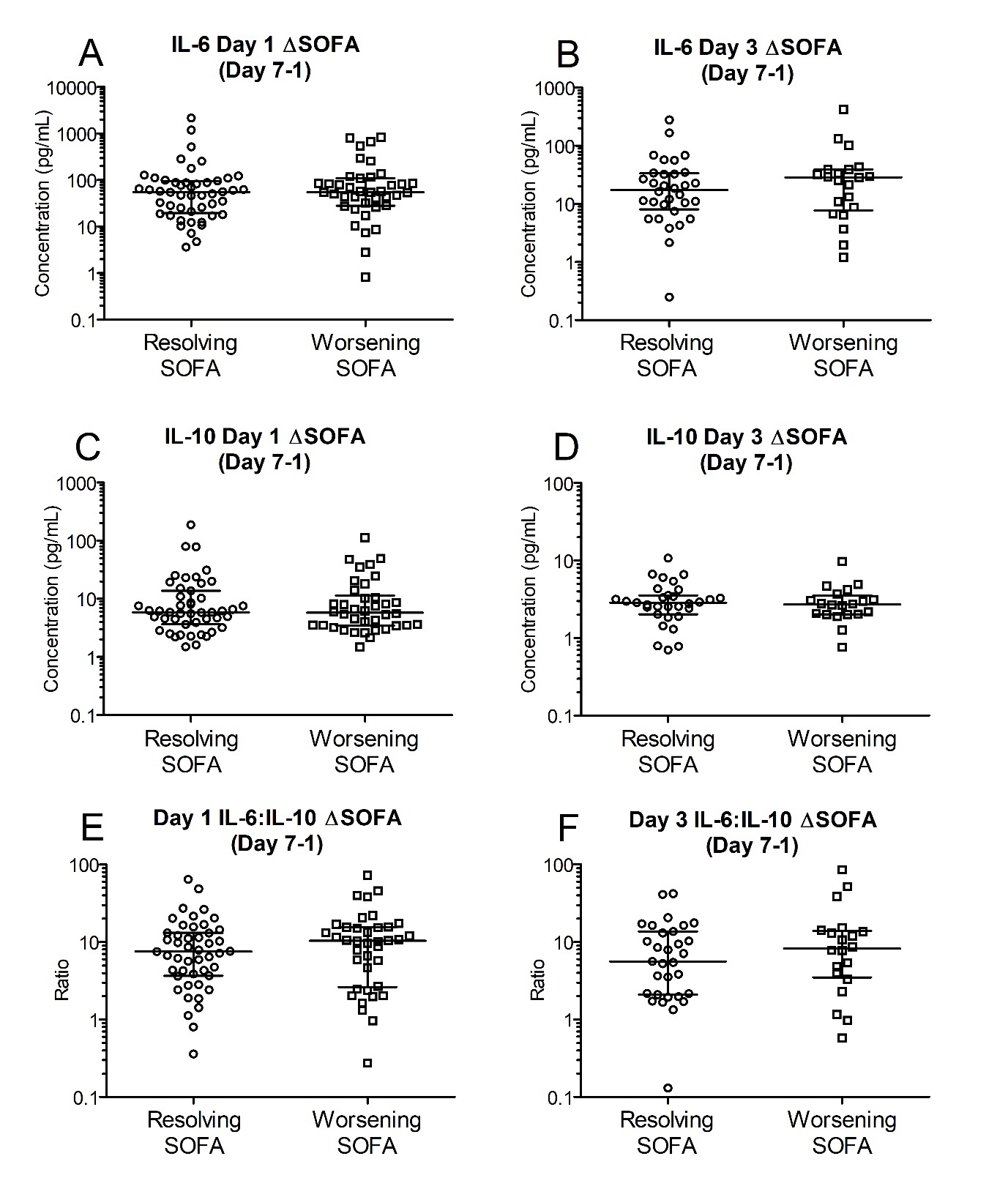
Supplementary Figure 5. Early (day 1 and 3) IL-6, IL-10, and IL-6:IL-10 ratios in the two groups based on ΔSOFA (SOFA _Day 7_-SOFA _Day 1_).**

Patients were retrospectively categorized according to their ΔSOFA, a calculated change in SOFA value (SOFA_Day7_ – SOFA_Day1_ score), creating two patient groups: ΔSOFA <0 (resolving condition) and ΔSOFA ≥0 (worsening condition). **A-B.** IL-6 levels on day one (56.17 [20.16-92.76] Vs 54.83 [30.22-97.50] pg/mL; n = 89, *P* =0.904) and day three (17.37 [9.18-32.94] Vs 28.58 [8.83-38.94] pg/mL; n =52, *P* = 0.506) did not differ between the two groups. **C-D.** IL-10 levels on days one (5.87 [3.78-12.36] Vs 5.77 [3.51-10.47] pg/mL; n = 89, *P* =0.757) and three (2.86 [2.22-3.51] Vs 2.72 [2.03-3.29] pg/mL; n = 52, *P* =0.734) were also not significantly different between the two groups. **E-F.** IL-6:IL-10 ratios on days one (7.51 [3.78-13.14] Vs 10.36 [3.19-15.46]; n = 87, *P* =0.411) and day three (5.60 [2.13-13.37] Vs 8.22 [3.86-13.70]; n = 52, *P* =0.282) were not significantly different between the two groups. All data expressed as median [Interquatiles].

**
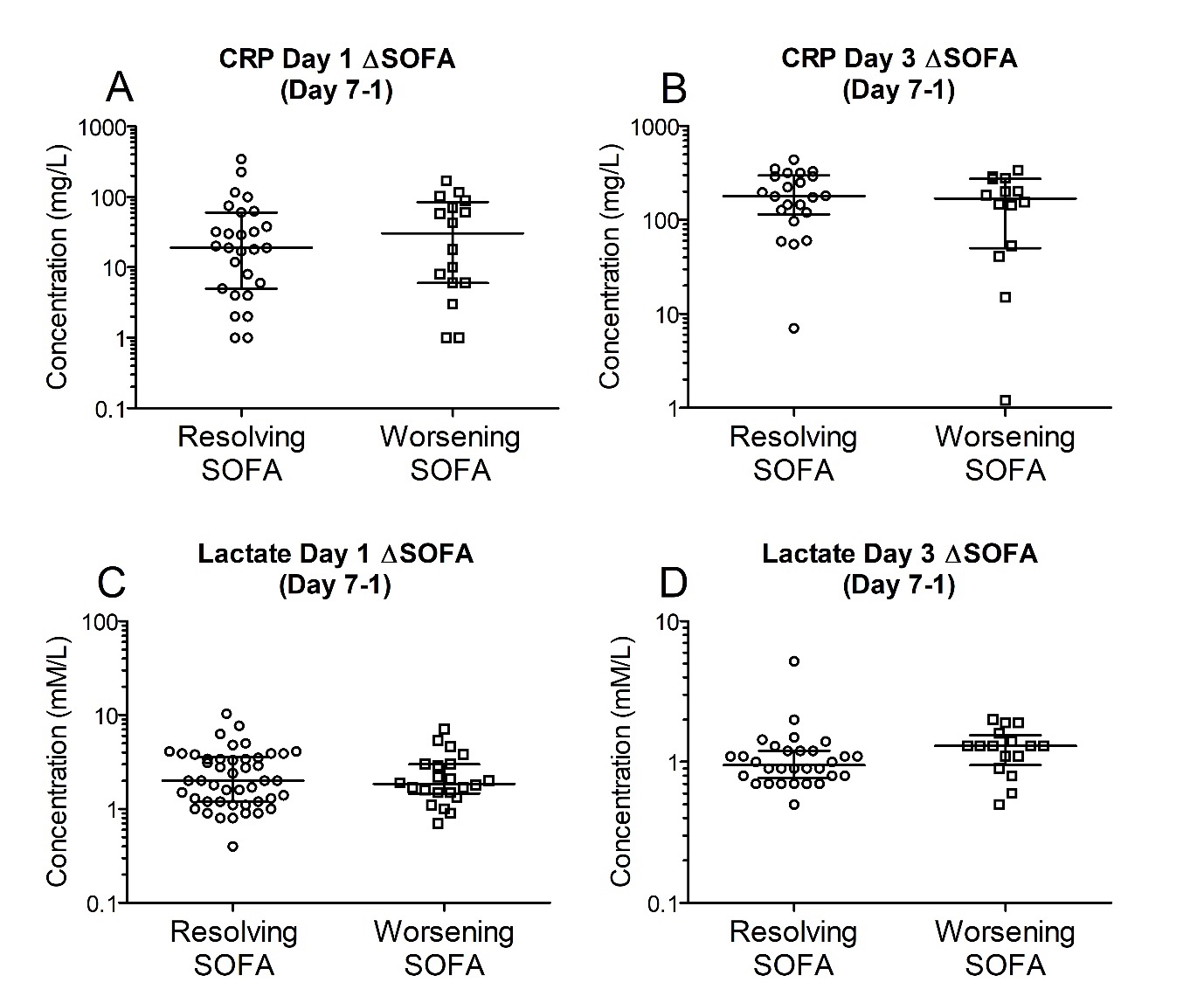
S****upplementary Figure 6. Early (day 1 and Day 3) C-reactive protein and serum lactate levels in blood in the two groups based on ΔSOFA (SOFA _Day 7_-SOFA _Day 1_).**

Patients were retrospectively categorized according to their ΔSOFA, a calculated change in SOFA (SOFA_Day7_ – SOFA_Day1_ score), creating two patient groups: ΔSOFA <0 (resolving condition) and ΔSOFA ≥0 (worsening condition). **A-B.** C-reactive protein levels at days 1 and 3 were not significantly different between the two groups. **C-D.** Serum lactate levels at days 1 and 3 were also not significantly different between the two groups.


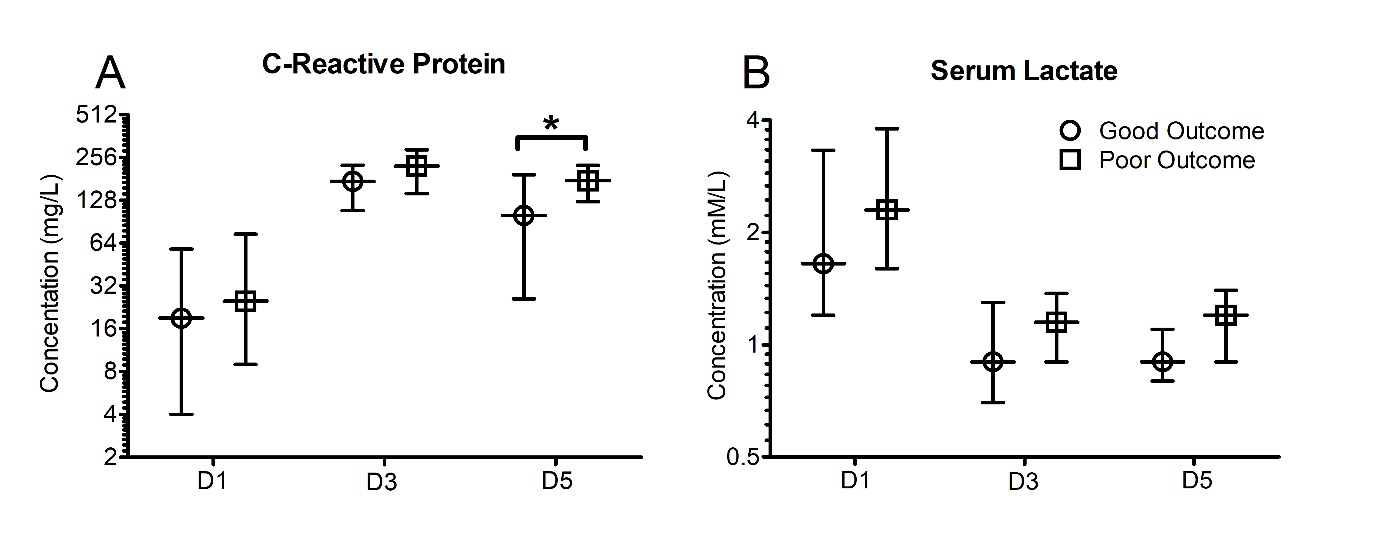
**Supplementary Figure 7. Early (Day 1, 3 and 5) C-reactive protein and serum lactate poorly predict patient outcome when stratified based on outcome five days post trauma.**

Patients were stratified based on good and poor outcomes based on their SOFA scores five days post trauma. Good outcome was classified as a SOFA score of <2, whilst poor outcome was classified as a SOFA score ≥2 five days post traumatic injury or death. **A.** C-reactive protein levels on day 5 were significantly greater in the poor group. **B.** Serum lactate concentrations were not significantly different between the groups. All data presented as median [Interquartiles]. Statistical significance was determined using Mann-Whitney U tests. * = *P* <0.05.

**
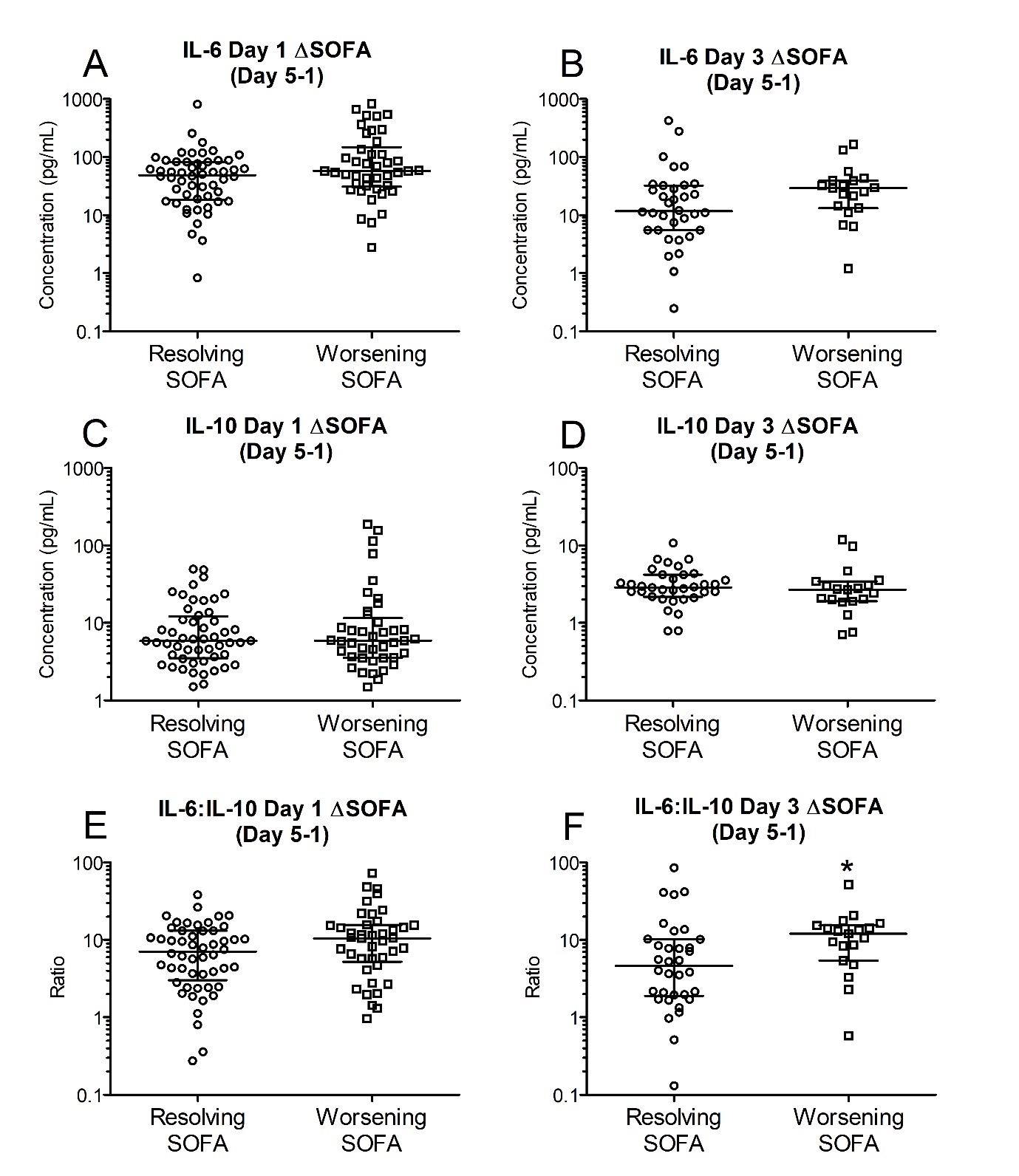
 Supplementary Figure 8. Early** **(Day 1 and 3) IL-6, IL-10, and IL-6:IL-10 ratios in the two groups based on ΔSOFA at day 5 (SOFA _Day 5_-SOFA _Day 1_)**

Patients were retrospectively categorized according to their ΔSOFA (SOFA_Day5_ – SOFA_Day1_ score), creating two patient groups: ΔSOFA <0 (resolving clinical status) and ΔSOFA ≥0 (worsening clinical status). **A-B.** IL-6 levels on days 1 & 3 were not different between the two groups (Day 1: 48.93 [19.58-82.14] Vs 57.58 [32.17-129.94] pg/mL; n = 89, *P* =0.077); Day three: (11.71 [5.50-31.27] Vs 29.14 [13.83-39.20] pg/mL; n = 54, *P* =0.065). **C-D.** IL-10 levels on days 1&3 were not significantly different between the two groups (Day 1: 5.87 [3.01-10.57] Vs 6.68 [3.51-12.53] pg/mL, (n = 89, *P* =0.960); Day 3: 2.82 [1.93-3.42] Vs 2.69 [2.03-4.19] pg/mL (n = 54, *P* =0.278). **E-F.** The IL6:IL10 ratios on days one (7.11 [3.42-13.11] Vs 10.49 [5.74-15.49]; n = 87, *P* =0.0758) was not significantly different between the groups. IL6:IL-10 ratio on day 3 however was significantly greater in the group showing clinical deterioration (4.65 [1.95-9.77] Vs [6.88-14.73]; n = 52, *P* =0.014). Data presented as median [Interquatiles]. Statistical significance was determined using a Mann-Whitney U test. * = *P* <0.05.

**
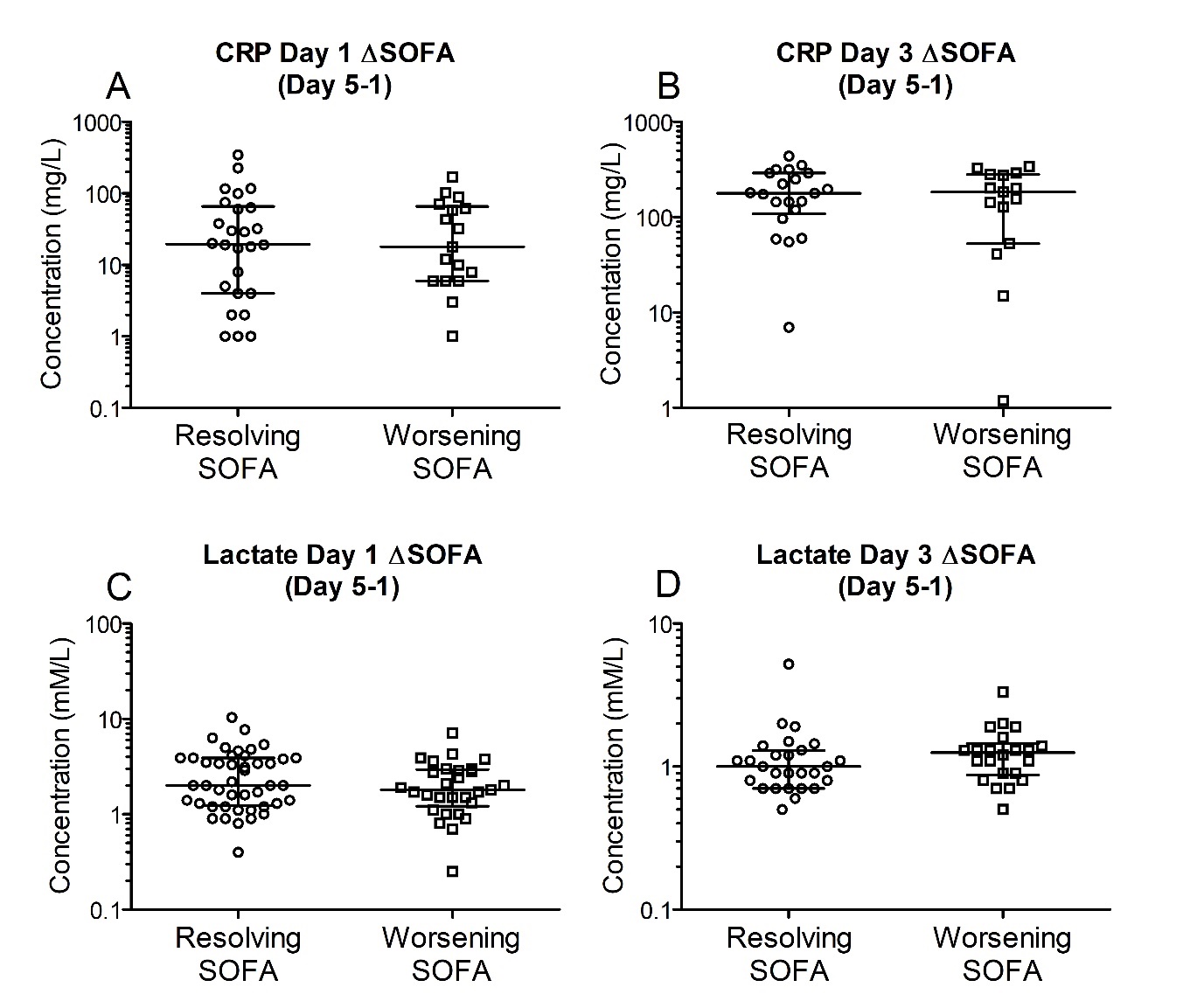
Supplementary Figure 9. Early (Day 1 and 3) C-reactive protein and serum lactate levels were unable to differentiate between resolving and worsening patients.**

Patients were retrospectively categorized according to their ΔSOFA (SOFA_Day5_ – SOFA_Day1_ score), creating two patient groups: ΔSOFA < 0 (resolving clinical status) and ΔSOFA ≥ 0 (worsening condition). **A-B.** C-reactive protein levels at days 1 and 3 were unable to differentiate between improving and deteriorating patient SOFA scores at day 5. **C-D.** Serum lactate levels at days 1 and 3 were unable to differentiate between improving and deteriorating patient SOFA scores at day 5.

## Supplementary Tables

# Supplementary Table 1. Patient characteristics and clinical parameters on admission in patients stratified on outcome five days following trauma.

| Variable | All Patients  (n=91) | SOFA < 2 on day 5 after admission  (n=53) | SOFA ≥ 2 on day 5 after admission or Death (n=38) | Odds Ratio  (95% CI) | *P* value |
| --- | --- | --- | --- | --- | --- |
| Age (Years) | 41.5 [29.0-60.8] | 39.3 [28.0-53.0] | 43.4 [29.5-64.0] | 1.01 (0.99-1.03) | 0.55 |
| Sex (Male) | 66 (72.5) | 36 (67.9) | 30 (78.9) | 1.56 (0.58-4.21) | 0.38 |
| HR (min^-1^) | 99.0 [13.0-112.0] | 101.0 [90.0-113.5] | 95.0 [76.0-107.0] | 0.99 (0.97-1.01) | 0.16 |
| SBP (mmHg) | 118.5  [98.8-144.0] | 110.0  [94.5-147.0] | 129.0  [106.0-142.5] | 1.01 (0.99-1.02) | 0.13 |
| DBP (mmHg) | 64.0 [53.0-77.0] | 63.0 [53.0-79.5] | 66.0 [53.0-77.0] | 1.01 (0.98-1.03) | 0.50 |
| GCS | 15.0 [6.0-15.0] | 15.0 [14.0-15.0] | 10.0 [3.0-15.0] | 0.84 (0.75-0.95) | 0.003** |
| FiO2 | 0.30 [0.21-0.52] | 0.31 [0.21-51.5] | 0.25 [0.21-0.65] | 1.00 (0.98-1.02) | 0.94 |
| PF ratio | 31.4 [7.3-49.4] | 39.2 [21.6-58.0] | 10.3 [3.8-34.2] | 0.99 (0.98-1.01) | 0.99 |
| WCC (x10^9^ L^-1^) | 14.4 [10.0-19.0] | 13.7 [10.0-18.8] | 15.4 [10.5-19.8] | 1.02 (0.99-1.04) | 0.19 |
| CRP (mg L^-1^) | 18.0 [6.0-60.5] | 19.0 [5.0-58.0] | 17.0 [6.8-72.0] | 1.00 (0.99-1.01) | 0.29 |
| Lactate  (mmol L^-1^) | 2.0 [1.4-3.7] | 1.8 [1.2-3.4] | 2.9 [1.7-4.8] | 1.20 (0.90-1.59) | 0.22 |
| ISS | 25.0 (9.0-41.0] | 23.5 [16.0-29.0] | 27.5 [15.5-38.75] | 1.04 (1.0-1.08) | 0.062 |
| APACHE II | 9.0 [6.5-12.0] | 9.0 [7.0-11.0] | 9.5 [3.3-14.0] | 1.05 (0.88-1.25) | 0.60 |
| SOFA | 4.5 [1.0-9.0] | 3.0 [1.0-5.0] | 8.0 [4.0-10.0] | 1.25 (1.10-1.42) | 0.001** |
| Antibiotics | 62 (68.1) | 41 (77.4) | 21 (55.3) | 0.36 (0.15-0.90) | 0.022* |
| IPPV | 42 (46.2) | 19 (35.8) | 23 (60.5%) | 2.74 (1.16-6.51) | 0.022* |

Data has been stratified according to SOFA scores five days post trauma and presented as median [interquartiles] and count (%). Odds ratio is presented with 95% confidence interval (CI). * = *P* <0.05, ** = *P* <0.01.
